# Antibodies for the Treatment of Brain Metastases, a Dream or a Reality?

**DOI:** 10.3390/pharmaceutics12010062

**Published:** 2020-01-13

**Authors:** Marco Cavaco, Diana Gaspar, Miguel ARB Castanho, Vera Neves

**Affiliations:** Instituto de Medicina Molecular, Faculdade de Medicina, Universidade de Lisboa, Av. Prof. Egas Moniz, 1649-028 Lisboa, Portugal; mcavaco@medicina.ulisboa.pt (M.C.); dianagaspar@medicina.ulisboa.pt (D.G.)

**Keywords:** adsorptive-mediated transcytosis, antibody fragments, blood–brain barrier, brain metastases, monoclonal antibodies, peptide shuttles

## Abstract

The incidence of brain metastases (BM) in cancer patients is increasing. After diagnosis, overall survival (OS) is poor, elicited by the lack of an effective treatment. Monoclonal antibody (mAb)-based therapy has achieved remarkable success in treating both hematologic and non-central-nervous system (CNS) tumors due to their inherent targeting specificity. However, the use of mAbs in the treatment of CNS tumors is restricted by the blood–brain barrier (BBB) that hinders the delivery of either small-molecules drugs (sMDs) or therapeutic proteins (TPs). To overcome this limitation, active research is focused on the development of strategies to deliver TPs and increase their concentration in the brain. Yet, their molecular weight and hydrophilic nature turn this task into a challenge. The use of BBB peptide shuttles is an elegant strategy. They explore either receptor-mediated transcytosis (RMT) or adsorptive-mediated transcytosis (AMT) to cross the BBB. The latter is preferable since it avoids enzymatic degradation, receptor saturation, and competition with natural receptor substrates, which reduces adverse events. Therefore, the combination of mAbs properties (e.g., selectivity and long half-life) with BBB peptide shuttles (e.g., BBB translocation and delivery into the brain) turns the therapeutic conjugate in a valid approach to safely overcome the BBB and efficiently eliminate metastatic brain cells.

## 1. Brain Metastases

Brain metastases (BM) account for significant morbidity and mortality. The exact incidence is unknown [1,2]. Based on various studies, investigators estimate that BM occurs in 10%–20% of adult patients with cancer [3]. Nevertheless, the incidence might be higher, and it is increasing due to prolonged life expectancy, increased resistance to cancer therapies, and improved imaging techniques. In addition, the increased patient survival by treating primary tumors may increase the number of patients that will develop more aggressive BM, or that are resistant to therapy. Among the different cancer types, lung cancer (19.9%), breast cancer (15.2%), and melanoma (6.9%) are the most common primary tumors developing BM [4]. After diagnosis, overall survival (OS) is poor. However, early diagnosis, improved systemic therapies, and multimodality treatments have significantly increased patients’ survival [5]. 

### 1.1. BM Pathophysiology

**Figure 1:**
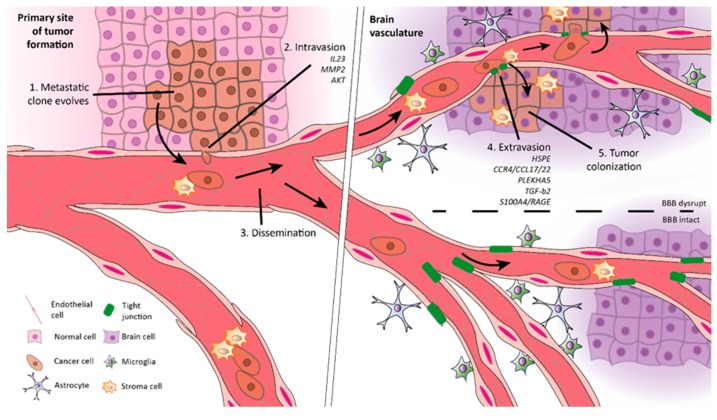
Steps in the formation of brain metastases (BM). Metastases formation begins in the microenvironment of the primary tumor with 1. metastatic clones developing, degrading the extracellular matrix (ECM), and suffering an epithelial–mesenchymal transition (EMT) to further detach from the connective tissue. 2. Subsequently, tumor cells invade and enter the circulation (intravation). 3. The dissemination within the vascular system drives tumor cells to distant sites, like the brain. 4. Then, they extravasate across the blood–brain barrier (BBB) and enter the brain parenchyma due to the release of proteolytic enzymes and cellular interactions. 5. Once inside the brain, cancer cells colonize the tissue and develop secondary tumors.

The pathophysiology of BM is complex and involves a multi-step process constituted of two major stages (Figure 1) [6]. The first stage is tumor migration, which includes (i) metastatic clone progression, due to tumor cells’ ability to degrade extracellular matrix (ECM); (ii) intravasation (transendothelial migration of cancer cells into vessels); (iii) dissemination (spread of tumor cells via bloodstream); (iv) extravasation (transendothelial migration of cancer cells into tissues). The second stage corresponds to tumor colonization.

The cells presented in the primary tumor are heterogeneous. Among others, the tumor microenvironment is composed of cancer stem cells (CSCs), partially differentiated progenitor cells, and fully differentiated end-stage cells [6]. Recent findings attribute to CSCs the primary responsibility for enhanced malignancy since they can complete the two stages of metastases formation (Figure 1) [7]. However, during cancer progression, other cells undergo an epithelial–mesenchymal transition (EMT), changing their plasticity by morphological and phenotypical conversions [8,9]. EMT enables non-CSCs to resemble a CSC state. Thus, they acquire the ability to invade and colonize distant sites, creating secondary niches that may progress to a secondary tumor [10]. Therefore, in the end, within the tumor microenvironment, all cells are malignant. Nevertheless, the development of distal metastases only occurs in <0.1% of disseminated cancer cells. Thus, although the formation of metastases represents a major threat, it is considered highly inefficient [8,11].

### 1.2. BBB Physiology

**Figure 2:**
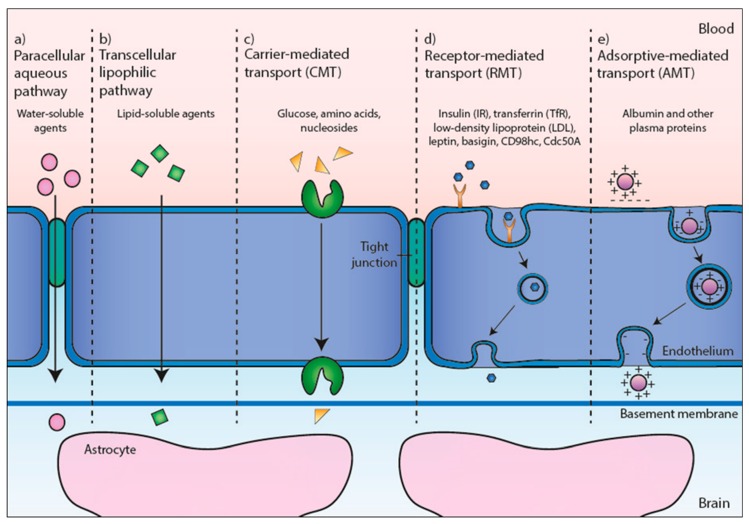
Pathways across the blood–brain barrier (BBB). Representation of the BBB formed by the endothelial cells and their interaction with astrocytes. Different translocation routes are presented. (**a**) Tight junctions usually restrict the penetration of water-soluble compounds. (**b**) The large surface area of the lipid membranes of the endothelium offers an efficient diffuse route for lipid-soluble agents. (**c**) Several transport proteins (carriers) are present in the endothelium for glucose (Gluc-1), amino acids, nucleosides, and other substances. (**d**) Large molecules such as antibodies, lipoproteins, proteins, and peptides can only transverse the BBB by receptor-mediated transport (RMT). (**e**) The transport of native plasma proteins or peptides is limited, but cationization can increase their uptake by adsorptive-mediated transport (AMT).

BBB is a complex system composed of a structurally distinct and continuous endothelial cell layer separating two brain compartments, namely, the blood and extracellular fluid. Its components include an endothelial cell layer, adjoined by tight cell-to-cell junction proteins, and pinocytic vesicles [12]. All together, they contribute to the selective permeability of the barrier, allowing brain homeostasis. The BBB is also dynamic. It responds to regulatory signals from both the blood and the brain [13], being the main portal into the brain of gaseous molecules, such as O_2_ and CO_2_, ions, nutrients, hormones, and water (Figure 2). Hydrophobic compounds (<500 Da) diffuse across the endothelium membrane. Carrier-mediated transport (CMT) is responsible for the transport of glucose and amino acid residues. While water-soluble molecules (e.g., ions) cross the BBB through ion channels. On the other hand, macromolecules (proteins and peptides) transport rely on endocytic vesicles, which involve either receptor-mediated transport (RMT) or adsorptive-mediated transport (AMT) [14,15].

Nevertheless, the BBB interacts with the metastatic cells in an unidentified way. The BBB is hypothesized to create a unique brain microenvironment and to influence metastatic colonization [16]. The increased permeability of tumor-associated endothelial cells, due to tumor penetration into the brain, permits leakage of proteins and water into brain parenchyma. The mechanism described is responsible for the edema often associated with BM [17]. Microglia and macrophages influence tumor proliferation and invasion by secreting multiple cytokines, growth factors, enzymes, and reactive oxygen species (ROS). Other immune cells may also participate in the BBB translocation. However, the exact mechanisms are debatable [18,19,20,21]. The BBB structure may be affected momentarily during cancer cells’ invasion; however, in other non-cancer-related central-nervous system (CNS) pathologies, only in advanced disease stages, the dysfunction is usually significant.

### 1.3. BM Treatment

Major developments have been made in understanding brain function, metastases progression, and the development of medical technologies. However, in many cases, the major drawback in BM treatment is the inefficient drug delivery into the brain [22,23]. The BBB remains the most significant obstacle to the efficient delivery of small-molecule drugs (sMDs) and therapeutic proteins (TPs) [24]. In addition, some authors also attributed to the therapeutic resistance of metastatic cells the responsivity for therapy inefficiency. According to them, during cancer progression, some survival pathways, such as the PI3K/AKT/mTOR are activated in specific cells, which contributes to the poor response of metastatic cells [25,26]. In the end, both mechanisms might be contributing to therapeutic failure.

The first-line approach to treat BM includes surgery, stereotactic radiosurgery (SRS), and whole-brain radiation therapy (WBRT) [27,28,29]. However, both systemic and intracranial disease control are also possible with the improved systemic therapies that have begun to offer greater potential for specific cancer types and genotypes. Thus, the management of BM has become increasingly individualized [1]. Depending on the histology and systemic disease status, physicians may consider all the available therapies. For instance, recommendations suggest better disease management by using a multidisciplinary modality in patients with BM from breast cancer, melanoma, and specific genotypes of non-small cell lung cancer (NSCLC) (e.g., epidermal growth factor receptor (EGFR) gene mutations, translocations in the anaplastic lymphoma kinase (ALK) gene). Whenever possible, patients may enroll in clinical trials for a novel or existing therapy. Still, given the paucity of effective treatment options, BM elimination represents an unmet clinical need [30].

## 2. Strategies to Overcome the BBB

The BBB restricts the delivery of therapeutics to the brain. Overall, 98% of sMDs and probably all TPs cannot cross the barrier by free diffusion [31,32,33]. In the last decade, intense investigation allowed the discovery of new strategies to increase brain penetration of existing therapeutics (invasive, pharmacological, and physiological) [34,35,36]. Ideally, the translocation should not compromise the BBB integrity. However, some of the current strategies do not meet this criterion, namely, the invasive and pharmacological approaches.

### 2.1. Invasive Approach

This strategy allows drugs to flow directly from the systemic circulation into the brain by BBB disruption using different methodologies. The most important are: (1) osmotic disruption, due to the administration of hypertonic solutions (e.g., mannitol) causing cells shrinking based on cell dehydration [37]; (2) ultrasound methods, which rely on transcranial delivery of low-frequency ultrasound waves resulting in the opening of tight junctions [38]; and (3) pharmacological agents, such as bradykinin-like compounds (e.g., histamine, bradykinin) that disrupt tight junctions by stimulating B_2_ receptors presented in endothelial cells and transiently increasing cytosolic Ca^2+^ [39]. The costs, anesthetic administration, and hospitalization are significant drawbacks for all these approaches. Also, the disruption of the BBB may increase tumor dissemination, as well as irreversible neuropathological changes due to the entry of unwanted substances [34].

### 2.2. Pharmacological Approach

The pharmacological approach relies on the observation that some molecules freely enter the brain owing to their molecular weight (<500 Da), charge (low hydrogen bonding capabilities), and lipophilicity [40]. Thus, researchers started modifying, through medicinal chemistry, molecules that are active against CNS diseases or BM to enable them to get into the brain [31]. Although it has enormous potential, the modifications may result in loss of pharmacological activity. In addition, the new molecule may become a substrate for the efflux pumps by increasing drugs’ lipophilicity, which decreases brain accumulation [34].

### 2.3. Physiological Approach

This natural strategy exploits the various transporters and receptors expressed at the BBB, as well as the physiological properties of the BBB (e.g., charge and lipid composition) (Figure 2) [41,42,43]. These translocation mechanisms are fundamental for the uptake of essential substances to maintain brain homeostasis. They can be classified into: (1) CMT, which are responsible for the cross of glucose (glucose transporter—GLUT1), amino acids (large neutral amino acid transporter—LAT1, and cationic amino acid transporter—CAT1), and nucleosides (nucleobase transporter—NBT); (2) RMT, fundamental for large molecules translocation, such as transferrin (transferrin receptor—TfR), insulin (insulin receptor—IR), low-density lipoprotein (lipoprotein receptor-mediated protein—LRP), leptin (leptin receptor—LEPR), and fragment crystallizable (Fc) fragment of immunoglobulin G (IgG) (Fc fragment of IgG receptor transporter α—FCGRT); and finally, (3) AMT that drives albumin and other plasma proteins to brain [44].

CMT is an interesting transporter due to the easy coupling of endogenous substrates to sMDs [45]. Besides, it is also possible to perform direct modification of sMDs to resemble CMT natural substrates. The changes allow drugs to be recognized and transported across the BBB [46]. Nevertheless, the molecules generated: (1) must mimic that of the endogenous CMT substrate; (2) should not affect CMT physiological function; and (3) must maintain its pharmacological activity. So far, targeting nanocarriers to CMTs have been the best example of the strategy’s success. However, the application of this approach was only possible for small molecules, as revised in Witt et al. [47].

Another promising strategy to develop molecules that can efficiently cross the BBB is the RMT. These molecules are known as Trojan Horses and can be either peptides or antibodies [48]. TfR and IR are the most important BBB receptors explored by researchers. Pardridge et al. have extensively documented the use of antibodies targeting these receptors [49,50,51]. The in vivo studies demonstrated an accumulation of different anti-TfR monoclonal antibodies (mAbs) in the brain tissue and a distinct biodistribution. The high affinity of antibodies towards these receptors is, however, a limitation since results in weak receptor dissociation. Consequently, the high-affinity antibodies follow the lysosomes pathway during intracellular trafficking leading to its degradation [52].

Yu et al. elegantly solved the problem by reducing the affinity of anti-TfR mAbs [53]. Next, the group developed a bispecific therapeutic antibody with a low affinity for TfR and a high affinity for the enzyme β-secretase (BACE1), an Alzheimer’s disease drug target. Relevant results were obtained from the evaluation of the bispecific antibody efficacy in non-human primates. The brain accumulation was significantly higher than control, and the amyloid β-peptide presence in the brain and serum reduced considerably [54]. Similarly to TfR, exciting studies targeting IR have been developed. Pardridge et al. have shown a total of 4% brain uptake 3 h after intravenous administration in Rhesus monkeys. In the treatment of Parkinson’s, stroke, metachromatic leukodystrophy, and Sanfilippo type A syndrome, some therapeutic drugs have been linked to the mAb and successfully translocated across the BBB [49,55].

Another interesting strategy to deliver drugs into the brain exploiting RMT is the use of nanoparticles (NPs) coupled with mAbs or peptides that recognize these receptors [43,56,57]. NPs are colloidal carriers of natural or synthetic origin with a size varying from 1 to 1000 nm. They are a fascinating system due to their modulating capacity concerning shape, size, hydrophobicity, coating, chemistry, and surface charge [58]. In addition, they also have a high capacity of drug payload, the relatively few mAbs or peptides to achieve high levels of drug targeting, protection of the encapsulated drug, and the ability to provide a controlled release of the drug [59,60].

Although the considerable achievements accomplished, the drawback of these RMT systems is related to the competition with natural substrates, which may affect brain homeostasis; and may result in receptors’ saturation due to the high affinity of antibodies [34,61,62]. To overcome these limitations, recently, more attention has been given to AMT. The concept of AMT through the BBB began with the observation that polycationic proteins’ brain uptake did not involve binding to the endothelial cell surface [63]. Electrostatic interaction between positively charged substances and negatively charged BBB drives the translocation. The vesicles created allow BBB cross and, consequently, brain accumulation [64]. Lack of selectivity of these systems and possible BBB disruption were the major concerns highlighted by researchers. However, the recent proof-of-concept given by the use of cell-penetrating peptides (CPPs) in BBB translocation (BBB peptide-shuttle) has launched a new interest in this strategy. These peptides have demonstrated a natural selectivity towards negatively charged membranes and the ability to translocate large cargoes without BBB damage both in vitro and in vivo models [65,66,67]. Therefore, the optimization of BBB peptide shuttles based-systems in the delivery of sMDs and TPs, using AMT, will be an area of intense investigation during the next decade.

## 3. Therapeutic Antibodies for BM Elimination

TPs are the standard of care in a number of therapeutic areas [68]. They are protein manufactured for biopharmaceutical use and include, for instance, mAbs, peptides, growth factors, cytokines, and enzymes [69]. Their production is relatively easy and relies mostly on either simple purification or recombinant DNA technology. Throughout the following sections, the description of antibodies’ activity and their therapeutic value only concern human antibodies. The activity of antibodies is species dependent. Thus, some features presented might not be accurate for non-human antibodies.

### 3.1. Monoclonal Antibodies

**Figure 3:**
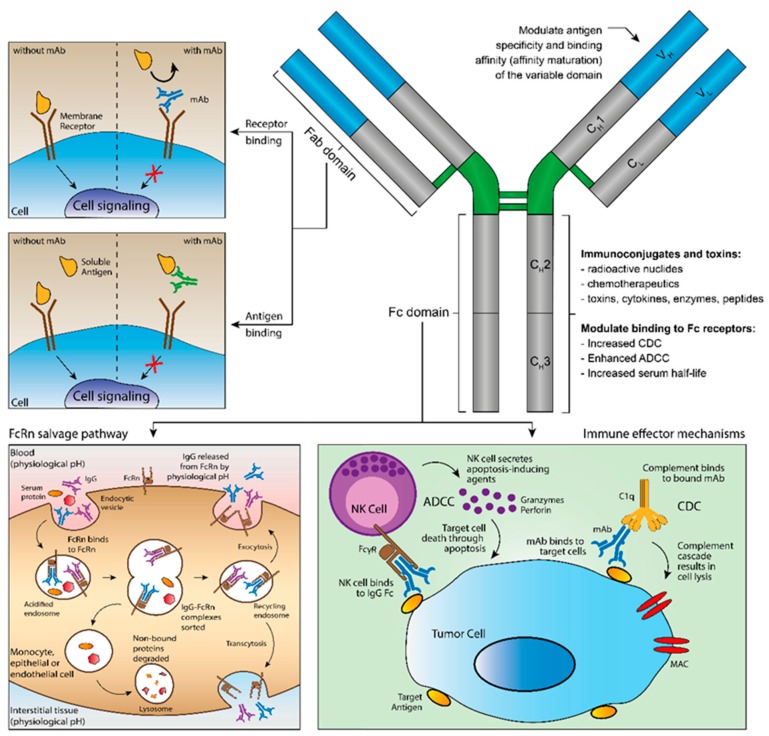
Main functions of therapeutic monoclonal antibodies (mAbs). mAbs have two antigen-binding fragments (Fabs) and one constant fragment crystallizable (Fc). The variable domain of the Fab confers specificity and binding affinity to either membrane receptors or soluble antigens. The Fc domain binds neonatal Fc receptor (FcRn), prolonging the half-life of mAbs; and connects immunoglobulin G (IgG) antibodies to immune effector mechanisms (antibody-dependent cell cytotoxicity—ADCC; and complement-dependent cytotoxicity—CDC) by engaging Fcγ receptors (FcγR) on immune cells, promoting cell lysis.

mAbs represent the fastest growing class of TPs. Currently, over 50 therapeutic antibodies are on the market [70]. They are complex molecules consisting of homodimers of variable and constant regions (Figure 3) [71]. The former has antigen specificity owing to the presence of complementary determining regions (CDRs). On the other hand, the Fc domain is responsible for the long half-life of antibodies, due to antibody recycling after interaction to the neonatal Fc receptor (FcRn) [72]; and immune activation (complement-dependent cytotoxicity—CDC; and antibody-dependent cellular cytotoxicity—ADCC) by engaging Fcγ receptors (FcγRs) on immune cells (e.g., neutrophils, natural killer cells, monocytes) [73]. High therapeutic tolerability and low risk-to-benefit ratios favor the use of therapeutic antibodies. Thus, their exquisite specificity, high binding affinity, long half-life, low toxicity, and versatility are characteristics that contributed to antibodies’ success [74,75]. Additionally, the low number of drug–drug interactions between mAbs and sMDs increased their combination in many therapeutic regimens [70].

The mechanism of action of mAbs differs depending on the molecule engineered (Figure 3). They can target soluble mediators (e.g., cytokines) to inhibit their binding to receptors and, consequently, inhibit signaling; or they can target membrane receptors either inducing or antagonizing signaling (e.g., programmed-cell death ligant-1—PD-L1; or human epidermal receptor-2—HER2, respectively) [76]. In addition, the presence of the Fc domain allows immune stimulation (CDC and/or ADCC). CDC is related to complement activation. Complement is one of the first mediators of the immune response to pathogens and cells. After binding, antibodies activate the classical complement cascade. Thus, releasing cytokines (e.g., anaphylatoxins and opsonins) and forming the membrane attack complex (MAC), which lead to cell lysis and phagocytosis [77]. On the other hand, ADCC occurred due to the interaction of the Fc domain with FcγRs on effector immune cells (e.g., neutrophils—FcγRI, natural killer cells—FcγIIIA; or monocytes—FcγIIIB). After recognition of an antibody-coated target cell, effector cells engage the release of granzymes and perforins [78]. The consequence is cell death. The magnitude of the stimulation of either CDC or ADCC depends on the IgG subset (IgG 1–4). For instance, IgG2 and IgG4 do not activate both mechanisms. Therefore, they are designed primarily for signaling blockage. Oppositely, IgG1 and IgG3 strongly activate both CDC and ADCC. Owing to its short half-life due to a low FcRn affinity, IgG3 does not have the therapeutic value of other IgG subsets [79].

### 3.2. Therapeutic Value

**Figure 4:**
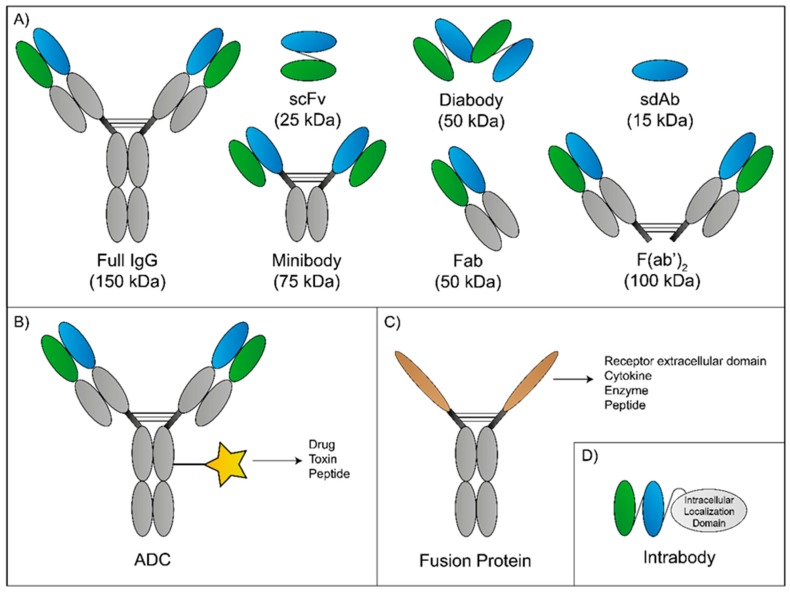
Novel or innovative monoclonal antibody (mAb) strategies. Schematic representation of different antibody formats currently in research. (**A**) An intact IgG molecule alongside with various antibody fragments and their respective molecular weight. (**B**) Antibody-drug conjugates (ADC) are usually intact IgG molecules linked to a drug, toxin, or peptide to increase the cargo selectivity. (**C**) Fusion proteins are biopharmaceutical molecules where the binding domains can be derived from a receptor extracellular domain, cytokine, enzyme, and peptide. Depending on the IgG molecule, the Fc region is capable of FcγR and C1q binding, potentially enabling the fusion protein to initiate antibody-dependent cell-mediated cytotoxicity (ADCC) and complement-dependent cytotoxicity (CDC). (**D**) The scheme shows an intrabody made of a variable region of the light and heavy chain constituting the antigen-binding domain and an intracellular location domain to allow nuclear binding.

The first mAb approved was muromonab-CD3 in the prevention of transplant rejection. Ever since, mAbs have been introduced in a number of therapeutic regimens in a wide range of conditions, such as organ transplantation (e.g., basiliximab and belatacept), inflammatory diseases (e.g., adalimumab and tocilizumab), and cancer (e.g., trastuzumab and cetuximab) [70]. The use of antibodies is increasing and improved mAb-based strategies will appear on the market in response to current therapeutic challenges (Figure 4). In particular, antibody research focused on the development of antibody fragments (e.g., single-chain Fv—scFv, single-domain antibody—sdAb, antigen-binding fragments—Fab); antibody–drug conjugates (ADC) (e.g., trastuzumab emtansine); fusion proteins (e.g., etanercept); and intrabodies [80,81,82]. Although physicians use mAbs in a variety of conditions, their applicability in the treatment of CNS diseases and BM remains challenging. Nevertheless, there are some mAb-based systems already approved (Table 1) or in investigation (Table 2).

### 3.3. CNS Diseases

Multiple sclerosis and episodic headache are the only neurologic pathologies where mAbs have been administrated, which started with the approval of natalizumab [83] and erenumab [84], respectively. Natalizumab is an IgG4 mAb targeting α4β1-integrin (very late activation antigen-4—VLA-4), which is present on the surface of leukocytes. After binding to VLA-4, the mAb inhibits the interaction between VLA-4 and vascular cell adhesion molecule-1 (VCAM-1). Consequently, reducing the adhesion, attachment, and migration of leukocytes across the BBB into the CNS [85]. In a pivotal phase III trial (AFFIRM), natalizumab reduced clinical relapse at one-year by 68% and the risk of continuous progression of disability by 42–54% over two years [86]. Nevertheless, the therapeutic effect observed occurs due to a peripheral action, instead of a direct antibody penetration into the brain. Erenumab is an IgG2, which targets the calcitonin gene-related peptide (CGRP) receptor [87]. The mAb competes with the binding of CGRP and inhibits its function at the CGRP receptor. The CGRP receptors are located at relevant sites to migraine pathophysiology, such as the trigeminal ganglion and the paraventricular structures. The BBB does not protect these regions. Thus, erenumab also exerts action at the periphery and not at the brain. In phase III STRIVE clinical trial, erenumab was able to significantly reduce the number of migraine days per month by 3.2 versus 1.8 in the placebo group. Efficacy was sustained up to one year [88].

A different strategy was applied in the management of Alzheimer’s disease. In this case, instead of antibodies acting at the periphery, researchers are using mAbs and re-engineered antibody fragments targeting natural brain portals (e.g., TfR and IR) [44]. Pardridge et al. reported for the first time an anti-β-amyloid (Aβ) scFv fused to mAbs targeting either TfR or IR [89]. The brain uptake of the molecule was of 0.88% ID/brain. The value is within the boundaries of the brain uptake of other drugs that are active in the brain [44,90,91]. However, the mAb demonstrated a high affinity towards the receptor. Thus, receptor dissociation was a major limitation. The consequence might be antibody degradation since the molecules, instead of crossing the BBB, follow the lysosomal pathway. Yu et al. elegantly solved the problem by reducing the affinity of these mAb [53]. In addition, the group further developed a bispecific therapeutic antibody with a low affinity for TfR and a high affinity for another Alzheimer’s disease drug target, the BACE1 [54]. The brain accumulation of the molecule in non-human primates was significantly higher than control, and the amyloid β-peptide presence in the brain and serum reduced considerably. The drawback of these RMT systems is the competition with natural substrates, which may affect brain homeostasis; and receptor saturation due to the high affinity of the antibodies engineered [44].

### 3.4. Brain Metastases

In the treatment of BM, the reality is different. In the recruitment phase of clinical trials, an active exclusion of patients presenting BM occurs [92,93]. Therefore, to date, no clinical trial supports the use of mAbs in the management of BM. The lack of information concerning efficacy and safety are the main reasons. Consequently, the standard cancer regimens with well-established antibody-based treatments cannot be applied to patients with BM [94]. Their use by physicians represents an off-label use. Nevertheless, a class of mAbs related to immunotherapy (e.g., nivolumab, pembrolizumab) is showing promising results. Several studies suggest their role in the elimination of metastatic tumors, such as in the brain. Still, more data is necessary to approve these antibodies in the treatment of metastatic brain cancers [95]. Another promising field is the radionuclide therapy. In the last years, numerous papers have been published reporting its success in brain tumors and metastases [96].

New therapeutic targets in metastatic progression in either primary or secondary tumors have driven intense research into the development of mAb-based systems [97,98], as they offer effective targeted treatment with low adverse events. However, the lack of specificity and poor BBB penetration render them ineffective. It is therefore imperative to find strategies that allow antibody translocation across the BBB. For instance, the use of antibody fragments to reduce their molecular weight. Or modify the mAbs to contain a translocation moiety, such as a CPPs. CPPs are effective in the delivery of large cargoes across cell membranes and even across the BBB (BBB peptide shuttles) [66,99]. Similar to the Trojan horse approach they engage interaction with BECs and BBB translocation, the main advantage being that CPP does not require receptors in the majority of the cases, thus reducing the toxicity of the system significantly.

## 4. The Role of BBB Peptide-Shuttles

CPPs are short peptides (less than 30 amino residues) capable of crossing cell membranes without causing significant membrane damage [99]. They represent a broad group of peptides with different physicochemical properties. Accordingly, they can be: (1) cationic, which comprises peptides with highly positive charges at physiological pH; (2) amphipathic, that contains both polar (hydrophilic) and nonpolar (hydrophobic) regions of amino acids; and (3) hydrophobic, the less studied class, which are CPPs mainly containing nonpolar residues, resulting in a low net charge. Naturally occurring proteins and peptides are the principal sources of CPPs [105,106], however, to optimize the peptides’ properties, fully engineered peptides have been designed, based on computational modeling [107].

The specific internalization mechanism of CPPs is unclear [108,109]. The peptide’s concentration, the cargo conjugated, the physicochemical properties, and molecular weight are features affecting the efficiency of cellular entry, as well as the internalization pathway followed. Nevertheless, energy-dependent endocytic pathways, which include clathrin-mediated endocytosis, caveolin-mediated endocytosis, and macropinocytosis, are considered the main translocation mechanisms [110]. The intensive research using peptides and the development of technologies that allowed their conjugation to TPs (e.g., recombinant DNA technology) resulted in the capacity of cargo-transportation not only across cell membranes, especially epithelia, but also the endothelial BBB (BBB peptide shuttle).

Human Immunodeficiency Virus Trans-activator of transduction (TAT) peptide was the first peptide demonstrating translocation properties [111]. Subsequently, many other peptides, like SynB, Penetratin, Angiopep-2, dNP2, and PepH3 were studied with relevant results [112]. Despite the variation in length and amino acids’ sequence, these peptides share common features. Among others, their amphipathic nature, net positive charge, theoretical hydrophobicity, helical moment, as well as the ability to interact with lipid membranes. The mechanism by which these peptide shuttles cross the BBB and mediate cargo translocation is not fully understood and may vary according to the concentration, cell type, and the cargo of interest [113]. Direct membrane permeation, RMT, and AMT are the three principal possibilities. The latter constitutes an advantage compared with others since it avoids enzymatic degradation, problems related to endosomal escape, receptor saturation, and toxicity, among others. The process is based on the electrostatic binding of positive charge peptide-shuttle to negative charge proteoglycans (Figure 2), forming a vesicle that transports the system across the endothelial cells layer [112].

The efficiency of large proteins delivery, such as antibodies or fusion proteins across cell membranes by CPPs, has been intensively studied, mainly in vitro. However, the delivery to the brain by peptide shuttles was not. Schwarze et al. performed the first in vivo study using BBB peptide shuttles. In their work, they successfully delivered a 120 kDa β-galactosidase fused to TAT into the brain [114]. These results showed that the direct delivery of proteins into the brain was possible. Afterward, others conjugated TAT to B-cell lymphoma-extra-large (TAT-Bcl-xL), glial cell-derived neurotrophic factor (TAT-GDNF), NR2B9c (TAT-NR2B9c), and c-Jun N-terminal kinase-1 (TAT-JNK1) fusion proteins, and evaluated their concentration in the brain [115]. In addition, the use of rabies virus glycoprotein (RVG) fused to brain-derived neurotrophic factor (RVG-BDNF), and fibroblast growth factor-4 (FGF4) fused to suppressor of cytokine signaling-3 (FGF4-SOCS3) also validated the use of these peptide shuttles [116,117].

Angiopep-2 is a 19-amino acid peptide, derived from the Kunitz domain, which binds to LRP1 and efficiently penetrates the BBB via RMT. In the study performed by Demeule et al., the translocation of angiopep-2 in an in vitro BBB model was found to be seven-fold higher than of aprotinin, an LRP1 natural ligand with BBB translocation properties [118]. Furthermore, the apparent distribution of the peptide shuttle in vivo was far greater than both transferrin and aprotinin, confirming the BBB translocation capabilities of angiopep-2. To further challenge the peptide, researchers conjugated it to an anti-HER2 mAb to investigate the ability of cargo translocation across the BBB. HER2+ breast cancer patients demonstrate a high incidence of BM. The low concentration of mAb in the brain provides a “sanctuary site” for tumor proliferation. Nevertheless, in this study, after carotid artery administration, 60% of the molecule was localized in the brain, demonstrating high brain accumulation. Besides, increased survival was reported compared with control [119].

dNP2 is an amphiphilic human-derived CNS-permeable peptide shuttle. To evaluate the abilities of cargo translocation across the BBB, Lim et al. conjugated the peptide with the cytoplasmic domain of CTLA4 (ctCTLA-4) [120]. CTLA-4 is an immune regulatory receptor expressed on the surface of T cells and often associated with susceptibility to multiple sclerosis. Thus, proving the autoimmune pathogenesis of the disease. In Lim et al. study, the administration of the dNP2-conjugated ctCTLA-4 protein successfully controlled autoimmune effector T-cell responses in an experimental autoimmune encephalomyelitis (EAE) model, an experimental mouse model of multiple sclerosis. The exact mechanism of BBB translocation and cell internalization were not determined. However, due to dNP2 properties, BBB crossing was considered to be AMT, and cellular uptake of the fusion protein through lipid-raft mediated endocytosis [120].

The most recent peptide shuttle reported is PepH3, a cationic peptide derived from Dengue virus type-2 capsid protein (DEN2C) [121]. In Neves et al. study, the in vitro BBB transmigration after 24 h was 67.2%. Furthermore, in an in vivo model, the peptide showed a brain biodistribution of 0.31% after 5 minutes. Although the exact mechanism is not fully described, studies with endocytosis inhibitors reveal that the PepH3 mechanism is consistent with the AMT. The peptide BBB translocation capacity was also evaluated in conjugation to anti-β-amyloid protein 42 (bAP42) sdAb (anti-bAP42 sdAb) [122]. bAP42 is an amyloid precursor protein fragment that plays a significant role in the formation of “senile plaques” characteristic of Alzheimer’s disease. Through conjugation of PepH3 to an antibody fragment, the investigators expected the increase in antibody concentration in the brain, followed by binding to bAP42, decreasing plaques formation. Also, the complex may be detected in peripheral blood aiding in the diagnosis of disease. Interestingly, the PepH3-anti-bAP42 sdAb conjugate showed a brain accumulation of 1.5% after the same period (5 minutes), showing that cargoes do influence the translocation mechanisms of BBB peptide shuttles [122].

The results obtained with these fusion proteins are promising. Nevertheless, their high elimination rates prevent the clinical use of such peptide shuttles-based systems. Peptides and small proteins are rapidly metabolized by serum/tissue proteases and easily eliminated by glomerular filtration [123]. Since they do not present a high circulation time, BBB translocation is also affected. By increasing circulation time, the brain concentration the conjugates will improve, due to a higher contact frequency and interaction time. Thus, increasing the half-life of such systems will hypothetically augment brain accumulation. Several strategies are available. The most interesting methods explore the human serum albumin protein (HSA) and the FcRn mediated recycling [124].

In the peptide shuttles-based approaches mentioned, researchers used mainly antibody fragments of a full IgG as therapeutic agents [120,122]. However, antibodies are proteins also used as pharmacokinetic enhancers owing to their Fc region. Thus, a strategy combining these properties with the translocation capabilities of peptide shuttles will improve the half-life of the system and increase brain accumulation. Depending on the target selected, the strategy may be used to eliminate BM or CNS diseases. Since antibodies are large proteins and BBB translocation is complicated, instead of a full antibody, the engineering of a minibody comprising the antibodies’ minimal domains (Fc fragment and variable region) to keep the antibody properties is preferable. In this, a reduction from 150 kDa for 25–80 kDa is achieved (Figure 4). These molecular weights are similar to the fusion proteins studied, increasing the interest of the strategy proposed. Another approach is the nanobodies (smallest Ab-derivative) which reveal promising results in preclinical and clinical studies in the elimination of brain tumors [102,125,126]. 

## 5. Conclusions and Future Perspectives

Our understanding of cancer biology and technological advances in cancer diagnosis and therapy improved significantly over the past decades. One of the landmarks for reversing the worldwide increase in cancer incidence and mortality was the development of more effective, tumor-specific, and less toxic anti-cancer drugs. The use of targeted therapy of human cancers using mAb-based systems has revolutionized cancer therapy. They are currently being used as the first choice to treat some of the most frequent metastatic cancers, such as HER2+ breast cancers or colorectal cancers. More recently, the efficacy demonstrated by antibodies inhibiting immune checkpoints has extended their use in other tumor types. In addition, they have also been introduced in many therapeutic protocols in combination with sMDs. For the treatment of cancer, the Food and Drug Administration (FDA) and the European Medicines Agency (EMA) have approved 32 mAbs-based systems. Interestingly, between 2012 and 2017 the number has doubled. Therefore, the value of mAbs in cancer therapy is undisputable.

Despite the excitement of mAbs-based systems in cancer treatment, their use in brain cancers and BMs are limited since they are unable to cross the BBB. Their molecular weight and hydrophilic nature difficult brain accumulation. Therefore, to create BM specific mAbs-based systems, they need to be modified to get across the BBB. Many strategies have been employed for mAbs to become a reality in the treatment of BMs. The most promising uses BBB peptide shuttles conjugated to mAbs-based systems. These peptides use endogenous routes, such as RMT or AMT to cross the BBB. Although RMT has been by far the most exploited route, it presents several disadvantages, namely, the receptor’s saturation and natural ligand competition. Therefore, with the advent of many peptide shuttles exploring AMT, the crossing of the BBB without interfering with brain homeostasis has become a reality. Consequently, the number of studies involving BBB peptide shuttles mAb-based systems are increasing in the literature.

In conclusion, BBB peptide shuttles mAb-based systems are being designed and studied with some limitations. However, the attractive results within different studies validate their application. Consequently, in the near future, it is expected a significant increase in the number of molecules conjugated to BBB peptide shuttles pushing the use of antibodies for the treatment of BMs into a reality.

## Figures and Tables

**Table 1:** Antibody therapeutics that are approved or in review for marketing for cancer or central-nervous-system (CNS) diseases.

Name	Brand Name	Type	Target	First Indication Approved or Reviewed	Approval Status (EU|USA)
[fam-]trastuzumab deruxtecan	-	Humanized IgG1 ADC	HER2	HER2+ metastatic breast cancer	N.A.|In review
Ado-trastuzumab emtansine	Kadcyla	Humanized IgG1 ADC	HER2	Breast cancer	2013|2012
Alemtuzumab	Lemtrada	Humanized IgG1	CD52	Multiple sclerosis; chronic myeloid leukemia#	2013; 2001 #|2014;2001 #
Atezolizumab	Tecentriq	Humanized IgG1	PD-L1	Bladder cancer	2017|2016
Avelumab	Bavencio	Human IgG1	PD-L1	Merkel cell carcinoma	2017|2017
Bevacizumab	Avastin	Humanized IgG1	VEGF	Colorectal cancer	2005|2004
Blinatumomab	Blincyto	Murine bispecific tandem scFv	CD19, CD3	Acute lymphoblastic leukemia	2015|2014
Brentuximab vedotin	Adcentris	Chimeric IgG1 ADC	CD30	Hodgkin lymphoma, systemic anaplastic large cell lymphoma	2012|2011
Cemiplimab	Libtayo	Human mAb	PD-1	Cutaneous squamous cell carcinoma	2019|2018
Cetuximab	Erbitux	Chimeric IgG1	EGFR	Colorectal cancer	2004|2004
Daclizumab	Zinbryta; Zenapax	Humanized IgG1	IL-2R	Multiple sclerosis #; prevention of kidney transplant rejection #	2016 #; 1999 #|2016 #; 1997 #
Daratumumab	Darzalex	Human IgG1	CD38	Multiple myeloma	2016|2015
Dinutuximab	Unituxin	Chimeric IgG1	GD2	Neuroblastoma	2015|2015
Durvalumab	IMFINZI	Human IgG1	PD-L1	Bladder cancer	2018|2017
Edrecolomab	Panorex	Murine IgG2a	EpCAM	Colon cancer	1995 *#|NA
Elotuzumab	Empliciti	Humanized IgG1	SLAMF7	Multiple myeloma	2016|2015
Enfortumab vedotin	-	Human IgG1 ADC	Nectin-4	Urothelial cancer	N.A.|In review
Eptinezumab	-	Humanized IgG1	CGRP	Migraine prevention	N.A.|In review
Erenumab	Aimovig	Human IgG2	CGRP	Migraine prevention	2018|2018
Fremanezumab	Ajovy	Humanized IgG2	CGRP	Migraine prevention	2019|2018
Galcanezumab	Emgality	Humanized IgG4	CGRP	Migraine prevention	2018|2018
Gemtuzumab ozogamicin	Mylotarg	Humanized IgG4 ADC	CD33	Acute myeloid leukemia	2018|2017; 2000 #
Ibritumomab tiuxetan	Zevalin	Murine IgG1	CD20	Non-Hodgkin lymphoma	2004|2002
Idarucizumab	Praxbind	Humanized Fab	Dabigatran	Reversal of dabigatran-induced anticoagulation	2015|2015
Inotuzumab ozogamicin	BESPONSA	Humanized IgG4 ADC	CD22	Acute lymphoblastic leukemia	2017|2017
Ipilimumab	Yervoy	Human IgG1	CTLA-4	Metastatic melanoma	2011|2011
Isatuximab	-	Humanized IgG1	CD38	Multiple myeloma	In review|In review
Natalizumab	Tysabri	Humanized IgG4	α4 integrin	Multiple sclerosis	2006|2004
Nebacumab	Centoxin	Human IgM	Endotoxin	Gran-negative sepsis	1991 *#|N.A.
Necitumumab	Portrazza	Human IgG1	EGFR	Non-small cell lung cancer	2015|2015
Nivolumab	Opdivo	Human IgG4	PD1	Melanoma, non-small cell lung cancer	2015|2014
Obinutuzumab	Gazyva	Humanized IgG1 Glycoengineered	CD20	Chronic lymphocytic leukemia	2014|2013
Ocrelizumab	OCREVUS	Humanized IgG1	CD20	Multiple sclerosis	2018|2017
Ofatumumab	Arzerra	Human IgG1	CD20	Chronic lymphocytic leukemia	2010|2009
Olaratumab	Lartruvo	Human IgG1	PDGFRα	Soft tissue sarcoma	2016|2016
Panitumumab	Vectibix	Human IgG2	EGFR	Colorectal cancer	2007|2006
Pembrolizumab	Keytruda	Humanized IgG4	PD1	Melanoma	2015|2014
Pertuzumab	Perjeta	Humanized IgG1	HER2	Breast cancer	2013|2012
Polatuzumab vedotin	Polivy	Humanized IgG1 ADC	CD79b	Diffuse large B-cell lymphoma	In review|2019
Ramucirumab	Cyramza	Human IgG1	VEGFR2	Gastric cancer	2014|2014
Rituximab	MabThera	Chimeric IgG1	CD20	Non-Hodgkin lymphoma	1998|1997
Sacituzumab govitecan	-	Humanized IgG1	TROP-2	Triple-negative breast cancer	N.A.|In review
Tafasitamab	-	Humanized IgG1	CD19	Diffuse large B-cell lymphoma	N.A.|In review
Tositumomab-l131	Bexxar	Murine IgG2a	CD20	Non-Hodgkin lymphoma	N.A.|2003 #
Trastuzumab	Heceptin	Humanized IgG1	HER2	Breast cancer	2000|1998

ADC, Antibody–drug conjugate; CGRP, Calcitonin gene-related peptide; CTLA-4, Cytotoxic T-lymphocyte-associated protein 4; EGFR, Epidermal growth factor receptor; EpCAM, Epithelial cellular adhesion molecule; Fab, Fragment antigen-binding; HER2, Human epidermal growth factor receptor-2; mAb, Monoclonal antibody; PD1, Programmed cell death protein-1; PDGFR, Platelet-derived growth factor receptor; PD-L1, Programmed death-ligand 1; scFv, Single-chain fragment variable; SLAMF7, Signaling lymphocytic activation molecule F7; TROP-2, Tumor-associated calcium signal transducer 2; VEGF, Vascular endothelial growth factor; N.A. Not approved; * Country-specific approval; # Withdrawn or marketing discontinued. Adapted from [100].

**Table 2:** Antibody therapeutics that are in investigation for cancer or central-nervous-system (CNS) diseases.

Name	Type	Target	Clinical Indications	Most Advanced Phase
(vic-)trastuzumab duocarmazine	Humanized IgG1 ADC	HER2	Breast cancer	Phase 3
[125I]-mAb 425	Human mAb	EGFR	Glioblastoma	Phase 2
[131I]-BC-2 mAb	Human mAb	Tenascin	Glioblastoma	Phase 1/2
[131I]-chTNT-1/B MAb	Human mAb	DNA-histone H1 complex	Glioblastoma	Phase 1/2
[131I]-SGMIB anti-HER2 VHH1	Humanized VHH	HER2	Breast cancer	Phase 1
[188Re]-labeled Nimotuzumab	Humanized mAb	EGFR	Glioblastoma	Phase 1
[211At]-labeled 81C6 mAb	Human mAb	Tenascin	Glioblastoma	Phase 1/2
131I-omburtamab	Murine mAb, radio-labeled	BT-H3	Neuroblastoma central nervous system/leptomenigeal metastases	Phase 2/3
68-Ga-NOTA-anti-HER2 VHH1	Humanized VHH	HER2	Brain metastases of breast carcinoma	Phase 2
ABBV-8E12	Human mAb	Tau protein	Alzheimer’s disease	Phase 2
Aducanumab	Human IgG1	Amyloid beta	Alzheimer’s disease	Phase 3
AL002	Human mAb	TREM2 receptor	Alzheimer’s disease	Phase 1
AL003	Human mAb	SIGLEC-3	Alzheimer’s disease	Phase 1
ALX-0651	Humanized VHH	CXCR4	-	Phase 1
Andecaliximab	Humanized IgG4	MMP9	Gastric or gastroesophageal junction adenocarcinoma	Phase 3
BAT8001	Humanized IgG1 ADC	HER2	Breast cancer	Phase 3
BCD-100	Human mAb	PD-1	Melanoma	Phase 2/3
Bernarituzumab	Humanized IgG1	FGFR2b	Gastric or gastroesophageal junction adenocarcinoma	Phase 3
BIB092	Human mAb	Tau protein	Alzheimer’s disease	Phase 2
biotin-coupled BC-4 + Avidin + [90Y]-Biotin	Human mAb	Tenascin	Glioblastoma	Phase 1/2
Bispecific nb-derived CAR-T cells	Bispecific Humanized tandem VH	CD19/CD20	Refractory/relapsed B-cell lymphoma	Phase 1
Camrelizumab	Humanized IgG4	PD-1	Hodgkin’s lymphoma, hepatocellular carcinoma	Phase 3
Carotuximab	Chimeric IgG1	Endoglin	Angiosarcoma	Phase 3
Crenezumab	Humanized IgG4	Amyloid beta	Alzheimer’s disease	Phase 3
CS1001	Human mAb	PD-L1	Non-small cell lung cancer	Phase 3
Depatuxizumab mafodotin	IgG1 ADC	EGFR	Glioblastoma	Phase 2b/3
Donanemab	Humanized IgG1 mAb	Amyloid beta	Alzheimer’s disease	Phase 2
Enfortumab vedotin	Human IgG1 ADC	Nectin-4	Urothelial cancer	Phase 3
Eptinezumab	Humanized IgG1	CGRP	Episodic migraines	Phase 3
Gantenerumab	Human IgG1	Amyloid beta	Alzheimer’s disease	Phase 3
I-131-BC8	Murine IgG1, radio-labeled	CD45	Ablation of bone marrow to hematopoietic cell transplantation in AML patients	Phase 3
IBI308	Human mAb	PD-1	Squamous cell non-small cell lung cancer	Phase 3
Isatuximab	Humanized IgG1	CD38	Multiple myeloma	Phase 3
JNJ-63733657	Human mAb	Tau protein	Alzheimer’s disease	Phase 1
KN035	mAb single domain	PD-L1	Bile tract carcinoma	Phase 3
L19IL2 + L19TNF	scFv conjugates	Fibronectin extra-domain B	Melanoma	Phase 3
Loncastuximab tesirine	Humanized IgG1 ADC	CD19	Diffuse large B-cell lymphoma	Phase 2
Margetuximab	Chimeric IgG1	HER2	Breast cancer	Phase 3
Mirvetuximab soravtansine	IgG1 ADC	Folate receptor 1	Ovarian cancer	Phase 3
Naxitamab	Humanized mAb	GD2	High-risk neuroblastoma and refractory osteomedullary disease	Phase 3
Opicinumab	Human mAb	LINGO-1	Multiple sclerosis, acute optic neuritis	Phase 2
Oportuzumab monatox	Humanized scFv immunotoxin	EpCAM	Bladder cancer	Phase 3
Polatuzumab vedotin	Humanized IgG1 ADC	CD79b	Diffuse large B-cell lymphoma	Phase 3
Relatlimab	Human mAb	LAG-3	Melanoma	Phase 2/3
Rovalpituzumab tesirine	Humanized IgG1 ADC	DLL3	Small cell lung cancer	Phase 3
Semorinemab	Humanized IgG4	Tau protein	Alzheimer’s disease	Phase 2
Solanezumab	Humanized IgG1 mAb	Monomers	Alzheimer’s disease	Phase 3
Spartalizumab	Humanized IgG4	PD1	Melanoma	Phase 3
Tisdelizumab	Humanized mAb	PD1	Non-small cell lung cancer, Hodgkin’s lymphoma	Phase 3
Trastuzumab deruxtecan	Humanized ADC	HER2	Breast cancer, HER2+ gastric or gastroesophageal junction adenocarcinoma	Phase 3
Tremelimumab	Human IgG2	CTLA4	Non-small cell lung, head & neck, urothelial cancer	Phase 3
TSR-042	Humanized mAb	PD1	Ovarian cancer	Phase 3
Ublituximab	Chimeric IgG1	CD20	Chronic lymphocytic leukemia	Phase 3
Utomilumab	Human IgG2	CD137	Diffuse large B-cell lymphoma	Phase 3
XMAB-5574	Humanized IgG1	CD19	Diffuse large B-cell lymphoma	Phase 2/3
Zagotenemab	Human mAb	Tau protein	Alzheimer’s disease	Phase 2
Zolbetuximab	Chimeric IgG1	Claudin-18.2	Gastric or gastroesophageal junction adenocarcinoma	Phase 3

ADC, Antibody–drug conjugate; AML, Acute myeloid leukemia; CGRP, Calcitonin gene-related peptide; CTLA-4, Cytotoxic T-lymphocyte-associated protein 4; CXCR4, Chemokine receptor type 4; DLL3, Delta-like protein 3; EGFR, Epidermal growth factor receptor; EpCAM, Epithelial cellular adhesion molecule; HER2, Human epidermal growth factor receptor-2; LAG-3, Lymphocyte-activation gene 3; mAb, Monoclonal antibody; MMP-9, Matrix metalloproteinase-9; PD1, Programmed cell death protein-1; PD-L1, Programmed death-ligand 1; scFv, single-domain fragment variable; TREM2, Triggering receptor expressed on myeloid cells 2. Adapted from [96,101,102,103,104].
